# Integrating attentional control theory and the strength model of self-control

**DOI:** 10.3389/fpsyg.2015.00824

**Published:** 2015-06-16

**Authors:** Chris Englert, Alex Bertrams

**Affiliations:** Department of Educational Psychology, Institute of Educational Science, University of Bern, Bern, Switzerland

**Keywords:** attentional control theory, anxiety, ego depletion, self-control, strength model of self-control

## Abstract

In the present article, we argue that it may be fruitful to incorporate the ideas of the strength model of self-control into the core assumptions of the well-established attentional control theory (ACT). In ACT, it is assumed that anxiety automatically leads to attention disruption and increased distractibility, which may impair subsequent cognitive or perceptual-motor performance, but only if individuals do not have the ability to counteract this attention disruption. However, ACT does not clarify which process determines whether one can volitionally regulate attention despite experiencing high levels of anxiety. In terms of the strength model of self-control, attention regulation can be viewed as a self-control act depending on the momentary availability of self-control strength. We review literature that has revealed that self-control strength moderates the anxiety–performance relationship, discuss how to integrate these two theoretical models, and offer practical recommendations of how to counteract negative anxiety effects.

## Integrating Attentional Control Theory and the Strength Model of Self-Control

Performing under high-pressure conditions along with the accompanying sensations of anxiety can impair academic (e.g., Zeidner, 1998; Ashcraft and Krause, 2007; Beilock, 2008) and perceptual-motor performance (e.g., Behan and Wilson, 2008; Nibbeling et al., 2012). One of the most influential theoretical frameworks that attempts to explain this finding is the attentional control theory (ACT; Eysenck et al., 2007).

The experience of anxiety often leads to rumination and worrisome thoughts regarding whether one can master a given situation or not, and what the potential consequences of failure may be (e.g., Eysenck, 1992). Worries preoccupy working memory capacity, reducing the accessibility of attentional resources used for storing and processing relevant information in the central executive (i.e., cognitive interference; e.g., Eysenck et al., 2005; for an overview on Baddeley’s model of working memory, see Baddeley, 1986, 2001). This load on working memory capacity can potentially influence performance during concurrent tasks for which efficient attention regulation is required (e.g., Ashcraft and Krause, 2007). Eysenck et al. (2007) proposed that anxiety and accompanying worries hinder efficient attention regulation by disrupting the balance between the top-down attentional system (which enables goal-oriented information processing) and the bottom-up attentional system (which allows for broader, stimulus-driven information processing; Corbetta and Shulman, 2002), leading to a dominance of the bottom-up stimulus-driven attentional system. However, top-down attentional control is necessary for superior performance in cognitively demanding tasks (e.g., complex mathematical operations; Ashcraft and Krause, 2007) and in fine perceptual-motor tasks (e.g., dart throwing; Nibbeling et al., 2012) because individuals need to inhibit their impulses (e.g., paying attention to task-irrelevant stimuli) and instead shift their focus onto the task at hand (for a broader discussion on the executive functions inhibition and shifting, see Miyake et al., 2000)^1^. The dominance of the bottom-up attentional system hampers the ability to focus on relevant stimuli (e.g., Carver and Scheier, 1981). This assumption is supported by studies in which visual attention, which is viewed as a reliable indicator of attentional control (Henderson, 2003), was negatively affected by increased anxiety levels (e.g., Behan and Wilson, 2008). To conclude, anxiety makes it harder to regulate attention volitionally.

Eysenck et al. (2007) have argued that anxious individuals are generally able to counteract the automatic tendency to process information in a bottom-up manner by investing additional effort (see also Nieuwenhuys and Oudejans, 2012). This claim has received support from studies in which there was no negative relationship between anxiety and performance (in academic settings: e.g., Seipp, 1991; in sports settings: e.g., Woodman and Hardy, 2003). However, ACT is not accurate in naming the actual processes that determine if anxiety impairs subsequent performance or not. Still, Eysenck et al. (2007) have argued that “[i]f auxiliary processing resources are available, impaired performance effectiveness is less likely to occur (…). If these resources are unavailable, then performance effectiveness will be impaired” (Eysenck et al., 2007, p. 337). This statement indicates that the ability to minimize the adverse anxiety effects seems to depend on the availability of some additional resource yet to be defined. In the present paper, we argue that the ability to exert self-control is the missing link in ACT (e.g., Baumeister et al., 1998).

## The Strength Model of Self-Control

In terms of the strength model by Baumeister et al. (1998), self-control is defined as a process in which predominant impulses can be volitionally overridden to achieve a specific goal, meaning the ability to resist immediate gratification in view of a more desirable outcome. Baumeister and colleagues have proposed that all acts of self-control—for instance, persistence (e.g., Baumeister et al., 1998), attention regulation (e.g., Schmeichel and Baumeister, 2010), or emotion regulation (e.g., Baumeister et al., 2000)—are based on one global metaphorical resource comparable to a muscle. But the capacity of this resource is limited, which means that one’s resource can become depleted after a primary act of self-control, and is not immediately replenished (ego depletion; e.g., Baumeister et al., 1998). In a state of ego depletion, decrements in self-control performance are more likely to occur. Most important for the present work is the finding that the ability to inhibit responses and to flexibly shift attention are also self-control acts that depend on the momentary availability of self-control strength (e.g., Ilkowska and Engle, 2010; Robinson et al., 2010; Hofmann et al., 2012). There are many studies that have supported this general finding, which is reflected in the findings of a recent meta-analysis that included 83 studies on ego depletion (Hagger et al., 2010; see Carter and McCullough, 2013, 2014, for criticism of this meta-analysis; see Hagger and Chatzisarantis, 2014, for a reply to the criticism).

## Integrating ACT and the Strength Model of Self-Control

According to Hagger (2009), theoretical integrations in general are beneficial for several reasons, including that they can fill theoretical gaps. Conforming to this notion, we aim to integrate the strength model of self-control into ACT. Such an endeavor is in line with very recent considerations of Hagger (2015) about the integration of the strength model into Hobfoll’s (1989) conservation of resources theory on coping with stress.

As stated above, increased levels of anxiety are associated with a tendency to worry about one’s performance (e.g., Eysenck, 1992). This may be problematic in tasks requiring top-down, goal-oriented information processing, highlighted by studies which have shown a negative anxiety–performance relationship (e.g., Ashcraft and Krause, 2007; Behan and Wilson, 2008; Beilock, 2008). As proposed in ACT, one can counteract this automatic tendency by investing additional effort or by activating additional resources (Eysenck et al., 2007; Nieuwenhuys and Oudejans, 2012). Speaking in terms of the strength model of self-control, counteracting predominant impulses—in this case, the automatic activation of bottom-up information processing under high levels of anxiety—is an ability that is dependent on self-control strength (e.g., Schmeichel and Baumeister, 2010). Therefore, we assume that self-control strength moderates the anxiety–performance relationship in tasks requiring goal-oriented attention regulation: In a state of ego depletion, an individual should display worse performance under high levels of anxiety because one cannot invest additional self-control strength to counteract the automatic tendency to process information in a bottom-up manner. When an individual has sufficient levels of self-control strength, we do not expect a negative anxiety–performance relationship because self-control strength may be serving as a shield against the negative anxiety effects on attention regulation. In the following, we report studies from the field of sport psychology (Englert and Bertrams, 2012, 2013; Englert et al., 2015a,b) and educational psychology (Bertrams et al., 2013; Bertrams and Englert, 2014) that support this claim.

## ACT and the Strength Model of Self-Control: Findings From Sport Psychology

For successful performance in fine perceptual-motor tasks (e.g., dart throwing), efficient attention regulation is needed (Abernethy et al., 2007): Irrelevant stimuli (e.g., the crowd) need to be blocked out, and instead, focus has to be shifted onto a relevant target (e.g., bull’s eye in darts; Nibbeling et al., 2012). However, increased levels of anxiety reduce the ability of athletes to concentrate on the respective targets; for instance, anxious individuals had shorter and fewer fixations on the bull’s eye in a dart-throwing task under anxiety (e.g., Nibbeling et al., 2012). In a series of studies, Englert and Bertrams (2012, 2013) were able to demonstrate that self-control strength moderates the anxiety–performance relationship in fine perceptual-motor tasks that depend on efficient attention regulation. Anxious participants in a state of ego depletion hit fewer basketball free-throws, scored lower in a dart-throwing task, and performed worse in a dexterity task compared to anxious participants with temporarily available self-control strength.

The assumption that ego depletion hinders efficient attention regulation in fine perceptual-motor tasks has received further empirical support from two recent studies. In the first study, participants performed a dart-throwing task in a high or low anxiety condition while wearing eye-tracking devices (Englert et al., 2015b). As previously mentioned, gaze behavior can be viewed as an indicator of attention regulation (e.g., Henderson, 2003; Vine and Wilson, 2011). Again, ego depletion moderated the anxiety–performance relationship because anxiety was only associated with performance impairments in the depletion condition. Additionally, depleted participants in the high-anxiety condition also showed less efficient gaze behavior because they displayed fewer and shorter fixations on the bull’s eye compared to non-depleted participants. Apparently, ego depletion hindered efficient attention regulation in anxious individuals.

In the second study, attention regulation in anxious individuals was also negatively affected by ego depletion (Englert et al., 2015a). Participants performed a basketball free-throw task under high-anxiety conditions while momentarily available self-control strength was experimentally manipulated. Additionally, they were listening to an external audio stream via stereo headphones representing worrisome thoughts often experienced in stressful situations (Oudejans et al., 2011). Again, anxiety was only associated with performance decrements in depleted participants. Interestingly, depleted participants also paid more attention to the distracting external audio stream compared to participants with temporarily available self-control strength. So, apparently, self-control strength protects attention regulation from increased distractibility under high levels of anxiety.

The aforementioned studies indicate that self-control strength moderates the anxiety–performance relationship in fine perceptual-motor tasks. In line with ACT (Eysenck et al., 2007), anxiety impaired attention regulation as increased distractibility was indicated by less efficient gaze behavior (Englert et al., 2015b) and by the tendency to pay more attention to irrelevant stimuli in anxious individuals (Englert et al., 2015a). Furthermore, as stated in ACT, individuals are generally capable of counteracting the negative anxiety effects on attention regulation. The presented studies show that individuals are only able to counteract the negative anxiety effects when they have sufficient self-control strength. If self-control strength is temporarily depleted, participants seem to be more prone to be distracted, leaving less attention available for the present task.

## ACT and the Strength Model of Self-Control: Findings From Educational Psychology

Plenty of research has demonstrated that anxiety can negatively affect cognitive performance (e.g., complex mathematical operations) and can thus be a negative predictor of one’s academic career (e.g., Zeidner, 1998). To perform the required cognitive operations in a cognitive task, working memory capacity is required (e.g., Eysenck et al., 2005). In a state of anxiety, though, internal factors (e.g., anxiety-related performance worries) or external factors (e.g., noise in the classroom) can consume parts of the limited resources of working memory, making it harder to adequately perform these cognitive operations (e.g., Deffenbacher, 1978). Nonetheless, there are also studies which do not report a negative statistical relationship between anxiety and cognitive performance (e.g., Seipp, 1991), leaving the question of which potential moderators could come into play. ACT has also been adopted to explain this inconsistent pattern of results: Just as in the case of sports performance, it is necessary to block out distracting stimuli to successfully finish a cognitive task. The inhibition function and the ability to shift attentional focus onto the immediate cognitive task can be impaired by heightened anxiety levels (e.g., Eysenck et al., 2007).

As previously mentioned, the ability to inhibit responses and to flexibly shift attention are self-control acts that depend on self-control strength (Ilkowska and Engle, 2010; Robinson et al., 2010; Hofmann et al., 2012). Consequently, there should be a stronger negative effect of anxiety on cognitive performance in individuals with depleted self-control strength compared to participants with available self-control strength. This hypothesis has received empirical support in two recently published papers.

Bertrams and Englert (2014) reported that there was no main effect of anxiety on knowledge retrieval in their study. However, there was a significant interaction between anxiety and self-control strength that revealed that there was only a negative effect of anxiety on knowledge retrieval in depleted participants. The authors concluded that depleted participants were not able to compensate for the negative anxiety effects on attention regulation. In the same vein, Bertrams et al. (2013) found a negative relationship between anxiety and performance in tests on verbal learning (Study 1) and in mental arithmetic tasks (Study 2) in ego depleted participants, whereas there was no significant relationship between anxiety and performance in participants with available self-control strength.

To summarize, in the reported studies, participants only suffered from anxiety in a state of ego depletion. Available self-control strength served as a buffer against the negative anxiety effects on cognitive performance. Boosting self-control strength may thus help anxious individuals to show their best possible performance in academic testing situations.

## Concluding Remarks and Potential Implications

In ACT it is assumed that anxiety leads to a dominance of the bottom-up stimulus-driven attentional system, which makes individuals more distractible, potentially impairing subsequent performance in tasks requiring selective attention (Eysenck et al., 2007). However, by initiating self-regulatory processes, anxiety-based effects on attention regulation can be compensated, but thus far, it had not been clear which self-regulatory processes are actually at work and why it is not always possible to compensate for the anxiety effects on attention regulation and performance. By integrating the strength model of self-control (e.g., Baumeister et al., 1998), we can now determine under which circumstances anxiety can potentially impair performance and under which conditions anxious individuals should be able to perform to their highest capabilities, thereby explaining the inconsistent findings on the anxiety–performance relationship (e.g., Seipp, 1991; Woodman and Hardy, 2003). Under ego depletion, individuals suffer from increased anxiety levels because they do not have the resources to offset the automatic tendency to process information in a bottom-up manner. If self-control strength is intact, efficient attention regulation and performance can be obtained (e.g., Englert and Bertrams, 2012, 2013; Bertrams et al., 2013; Englert et al., 2015a,b).

We also reviewed studies that focused on the assumed process—increased distractibility—which is responsible for performance decrements under high-anxiety levels and is mostly affected by ego depletion. According to ACT (Eysenck et al., 2007), anxiety leads to increased distractibility, making it harder to focus on the given task. Anxious participants with temporarily available self-control strength were not only able to keep their performance levels consistent, but were also more adept in regulating their attention because they displayed more efficient gaze behavior (Englert et al., 2015b) and paid less attention to irrelevant, distracting stimuli compared to anxious participants with depleted self-control strength (Englert et al., 2015a).

Recently, Inzlicht and Schmeichel (2012) argued that the ego depletion effect would be better explained by temporary motivational and attentional shifts toward reward and gratification. Moreover, Kurzban et al. (2013) suggested as an alternative explanation to the assumption of limited self-control strength that people disengage from self-control when they had experienced high costs relative to the benefits of exerting self-control in an initial task. These alternative accounts are, however, incompatible with the empirical data we reported in the present work: By reducing their self-control during test situations, anxious people do obviously nothing that would help them to achieve some sort of reward. Rather, they boost the likelihood of failure, and the experience of even higher costs. In contrast, the notion of a limited self-control capacity is well in line with the abovementioned findings.

We would also like to mention some ideas on how to prevent anxiety-related performance impairments. Self-control strength is often compared to a human muscle: Like a muscle, one’s self-control strength can become exhausted and depleted, impairing subsequent performance (e.g., Baumeister et al., 1998). Also like a muscle, one’s self-control strength can be trained, enabling better self-control performance over time (for an overview, see also Baumeister et al., 2006). For instance, participants that regularly exerted self-control over a 2-week period outperformed participants that did not receive self-control training (e.g., Gailliot et al., 2004; Muraven, 2010). Interestingly, there has been no study, to our knowledge, that has tested whether regular self-control training help anxious individuals to improve their performance under high-pressure situations. Therefore, future studies should try to transfer these laboratory-based findings to more applied contexts. In line with the muscle metaphor, there are also strategies which may lead to a quicker replenishment of self-control strength, as Tyler and Burns (2008) have demonstrated that active relaxation is a useful strategy in that regard. Transferring self-control training to applied settings with the aim of reducing the potential negative anxiety effects on performance might not be too difficult, or, in some cases, a transfer might even have taken place already. For instance, sport psychologists highly recommend the use of active relaxation techniques before and during sporting competitions (e.g., Williams, 2006). Also, in the classroom, relaxation techniques have been applied before important exams (e.g., Zeidner, 1998).

The above reviewed studies make a strong case for integrating the assumptions of the strength model of self-control (e.g., Baumeister et al., 1998) into ACT (Eysenck et al., 2007). Such an integration makes up for a major shortcoming of ACT because, thus far, it had not been clear which self-regulatory processes determine whether or not anxious individuals can counteract detrimental anxiety effects. By investing self-control strength, performance can be maintained despite high-anxiety levels.

Based on the present thoughts, it may also be possible to further integrate ACT into a broader theoretical context in future work. According to the default-interventionism framework of the dual-process theories (Morewedge and Kahneman, 2010; Evans and Stanovich, 2013), the so-called System 1 reacts to given situations by generating automatic default response tendencies. System 2 would intervene with controlled operations when these default responses run into difficulties. However, as Bertrams et al. (2015) recently argued, intervention of System 2 may fail when self-control strength is currently low. Relating this approach to ACT, this means that anxiety would cause automatic responses that could harm performance by default (i.e., bottom-up processing that causes a decline of attention from the task at hand). When self-control strength is available, however, one could intervene by controlled attention regulation.

## Author Contributions

CE substantially contributed to study design, and writing of the manuscript. AB contributed substantially to study design, and writing of the manuscript. Both authors approve the final version of the manuscript. The authors agree to be accountable for all aspects of the work in ensuring that questions related to the accuracy or integrity of any part of the work are appropriately investigated and resolved.

### Conflict of Interest Statement

The authors declare that the research was conducted in the absence of any commercial or financial relationships that could be construed as a potential conflict of interest.

## Footnotes

1 Apart from inhibition and shifting, Miyake et al. (2000) also identified updating as a third executive function of working memory. However, Eysenck et al. (2007) have argued that updating is an executive function which supposedly is not significantly affected by anxiety. Therefore, we have not focused on updating in this paper.
